# Incorporation of charge discreteness and ion correlations into lattice models of ionic liquids

**DOI:** 10.3389/fchem.2024.1502840

**Published:** 2024-12-05

**Authors:** Guilherme Volpe Bossa, Sylvio May

**Affiliations:** ^1^ Institute of Mathematical and Physical Sciences, Universidad Austral de Chile, Valdivia, Chile; ^2^ Department of Physics, North Dakota State University, Fargo, ND, United States

**Keywords:** ionic liquid, electrostatics, ion correlations, charge discreteness, mean-field, quasi-chemical approximation, random mixing approximation, boundary conditions

## Abstract

Lattice-based mean-field models of ionic liquids neglect charge discreteness and ion correlations. To address these limitations, we propose separating the short-range and long-range parts of the electrostatic interaction by truncating the Coulomb potential below a fixed distance that is equal to or slightly larger than that between neighboring ions. Interactions and correlations between adjacent ions can then be modeled explicitly, whereas longer-ranged electrostatic interactions are captured on the mean-field level. We implement this approximation into the framework of modeling a compact, solvent-free ionic liquid by, first, considering terms up to the fourth order of the operator that represents the truncated Coulomb potential and, second, by accounting for electrostatic correlations between pairs of neighboring ions on the level of the quasi-chemical approach. A set of boundary conditions for the resulting self-consistent fourth-order differential equation follows from functional minimization of the free energy. The differential capacitance of an ionic liquid in contact with a planar electrode is calculated analytically up to quadratic order in the electrode’s surface charge density by solving the linearized model and applying a perturbation approach valid beyond the linear regime. We demonstrate that charge discreteness enhances the differential capacitance, whereas electrostatic correlations between ion–ion pairs drive the transition from a bell-shaped to a camel-shaped profile of differential capacitance. Our approach offers a systematic way to further improve the treatment of charge discreteness, account for short-range electrostatic and non-electrostatic interactions, and include higher-order ion–ion correlations.

## 1 Introduction

Ionic liquids consist of mixtures of cations and anions, usually of asymmetric size, that can remain in a liquid phase at temperatures typically below 100°C (Hayes et al., 2015). After initially being described as “water-free salts” (Walden, 1914), ionic liquids continue to gain attention due to their unique properties, including low volatility, high thermal stability, and excellent solvating capabilities (Sowmiah et al., 2009; Eyckens and Henderson, 2019). Given the diversity of molecular structures that form ionic liquids (Hayes et al., 2015) and their potential applications in energy conversion and storage (Armand et al., 2009; Wishart, 2009; Torimoto et al., 2010; Watanabe et al., 2017), it is not surprising that optimizing function–structure relationships is both complex and highly rewarding (Silva et al., 2020; Wang et al., 2020; Khlyupin et al., 2023; Nesterova et al., 2025). The challenges extend even to the level of understanding underlying thermodynamic and dynamic properties of ionic liquids (Weingärtner, 2008; Silva et al., 2020; Nordness and Brennecke, 2020). Compared to the ions in ordinary electrolytes, those of ionic liquids are densely packed and thus subject to ion–ion correlations due to electrostatic and non-electrostatic interactions (Del Pópolo and Voth, 2004).

Classical mean-field modeling (Kornyshev, 2007; May, 2019; Goodwin et al., 2023), which is a powerful conceptual tool to characterize ions in a solvent at sufficient dilution, fails for ionic liquids. Consequently, significant efforts have been undertaken in the past to incorporate ion correlations due to electrostatic and non-electrostatic interactions into the modeling of ionic liquids (Kondrat et al., 2023). Examples include the use of field theory (Démery et al., 2012; Nakamura, 2015; Budkov, 2020; Chao and Wang, 2020), density functional theory (Wu et al., 2011; Henderson et al., 2011; Ma et al., 2014), a microscopic cluster expansion model (Avni et al., 2020), molecular dynamics (Wang et al., 2007; Fedorov and Kornyshev, 2008; Coles et al., 2020; Zeman et al., 2021; Ye and Wang, 2022) and Monte Carlo (Kondrat et al., 2011; Bhuiyan et al., 2012) simulations, as well as phenomenological extensions of mean-field approaches (Frydel, 2016; Goodwin et al., 2017; Downing et al., 2018a). To allow for oscillations in ionic concentration profiles, Bazant, Storey, and Kornyshev (BSK) proposed a phenomenological Landau–Ginzburg-like model that leads to a fourth-order modified Poisson–Boltzmann equation with a correlation length that accounts for short-range ion–ion interactions (Bazant et al., 2011). The BSK approach has been used widely (Yochelis, 2014; Shalabi et al., 2019; Varner and Wang, 2022; Nesterova et al., 2025) and linked to microscopic approaches (Blossey et al., 2017; Avni et al., 2020). Yet, understanding of the physical basis of higher-order Poisson–Boltzmann approaches and their relation to even the most basic molecular properties such as charge discreteness, ion polarizability, non-electrostatic interactions, and electrostatic ion–ion correlations remains incomplete.

The present work aims at incorporating both charge discreteness and electrostatic correlations between ion pairs into a lattice-based model of an ionic liquid that is in contact with a single planar electrode. To account for charge discreteness, we separate the short-range and long-range parts of the electrostatic interaction by truncating the Coulomb potential below a distance $r_{0}$ to the neighboring ions. The operator that results from this truncation involves an infinite number of higher-order derivatives, yet it becomes equivalent to the BSK model in the limit of small $r_{0}$ . We focus on this limit, which results in an approximate incorporation of electrostatic interactions on the level of continuum electrostatics except for nearest neighbors. In contrast, electrostatic interactions among nearest neighbor ions are modeled explicitly, thereby accounting for their discrete nature. To account for electrostatic correlations among neighboring ions, we adopt the quasi-chemical approximation (QCA) approach (Sher et al., 1987; Davis, 1996) when expressing the configurational entropy of the lattice gas. In principle, QCA can account for a correlation among any number of ions, but in the present work, we only include two-body correlations among nearest neighbor ions. Hence, we propose a method that accounts for both charge discreteness and electrostatic correlations between ion pairs in the modeling of ionic liquids. We recognize that the present work assumes $r_{0}$ is sufficiently small and neglects ion correlations that involve more than two ions. Yet, we also point out that, in principle, this approach can be extended to account for larger $r_{0}$ and higher-order correlations. In addition to presenting the method and deriving the self-consistency relationship (a fourth-order differential equation) plus its boundary conditions, we calculate analytic results for the differential capacitance up to quadratic order in the surface charge density of the electrode and numerical results for larger surface charge densities. Our results are compared with limiting cases, such as the absence of electrostatic correlations between ion pairs and the negligence of ion discreteness. We find that charge discreteness enhances the magnitude of the electrostatic potential, and thus also the differential capacitance, whereas electrostatic correlations between ion pairs induce a transition from a bell-shaped to a camel-shaped profile of the differential capacitance.

## 2 Theory

Consider a compact, solvent-free ionic liquid containing monovalently charged cations and anions of the same size. We represent the ionic liquid by a lattice of coordination number z , volume ν per lattice site, and lattice spacing $b∼\nu^{1/3}$ , with each site being occupied by either a cation or an anion. $\phi_{1}=\phi_{1}\left(r\right)$ and $\phi_{2}=\phi_{2}\left(r\right)$ denote the local mole fraction of cations (index “1”) and anions (index “2”), respectively, at position r . The absence of solvent is expressed by the condition $\phi_{1}+\phi_{2}=1$ at every position r . In the present work, we aim at separating contributions to the electrostatic energy of the ionic liquid that originate from the short-range and long-range parts of the Coulomb potential. To introduce this method, we write for the electrostatic energy due to the long-range part.

$$U=\frac{1}{2\nu^{2}}\int d^{3}r\int d^{3}r^{\prime }\;\eta \left(r\right)\;u\left(\left|r-r^{\prime }\right|\right)\;\eta \left(r^{\prime }\right),$$ (1)

where we have defined the difference $\eta =\phi_{1}-\phi_{2}$ in mole fractions between the cations and anions. Note that eη/ν specifies the local volume charge density, where e denotes the elementary charge, and that η is positive in regions with an excess of cations over anions. Equation 1 accounts for all pairwise interactions among the ions within the ionic liquid: u(r) for cation–cation and anion–anion pairs, and −u(r) for cation–anion and anion–cation pairs, given that the two ions are separated by a distance $r=|r-r^{\prime }|$ . We introduce a dimensionless potential Ψ=Ψ(r) through:

$$\Psi \left(r\right)=\frac{1}{\nu }\;\int d^{3}r^{\prime }\;\eta \left(r^{\prime }\right)\;u\left(\left|r-r^{\prime }\right|\right),$$ (2)

where, here and in the following, we express energies in units of $k_{B}T$ , where $k_{B}$ is the Boltzmann constant and T is the absolute temperature. In order to recast Equation 2 into a *local* equation for the potential Ψ , we introduce a yet unknown differential operator A such that Au(r)=−δ(r) , where δ(r) is the Dirac delta function. This indeed implies the local equation:

$$A\Psi =-\frac{\eta }{\nu },$$ (3)

and corresponding electrostatic energy

$$U=-\frac{1}{2}\int d^{3}r\;\Psi \;A\Psi .$$ (4)

Only after specifying a concrete interaction potential u(r) , we can determine the corresponding differential operator A . This is addressed in the following section.

### 2.1 Truncated Coulomb potential

**FIGURE 1:**
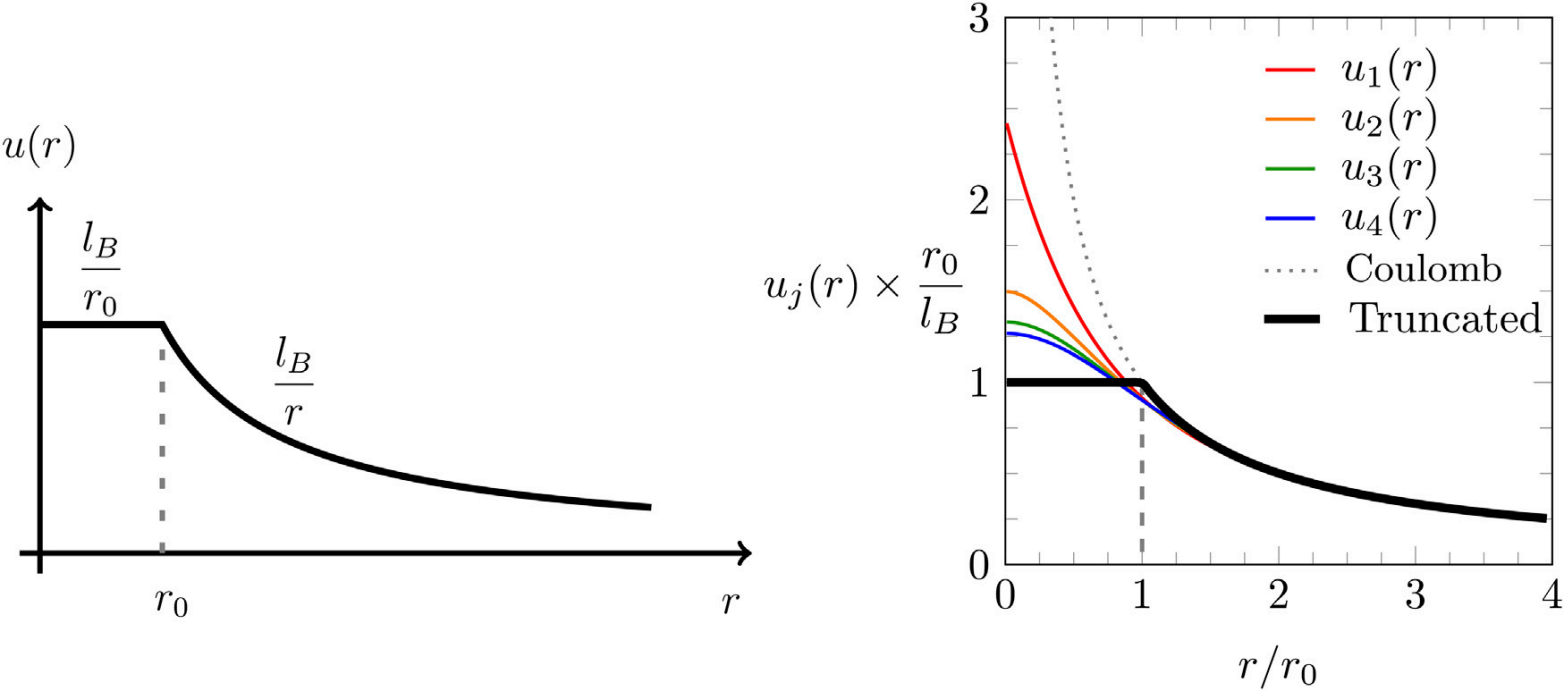
Left diagram: plot of the truncated Coulomb potential u(r) as a function of the radial distance r according to Equation 5. The truncation distance is $r_{0}$ , and the Bjerrum length is $l_{B}$ . Right diagram: truncated Coulomb potential u(r) (thick solid line) together with the increasingly more accurate approximations $u_{1}\left(r\right)$ (first order, shown in red color), $u_{2}\left(r\right)$ (second order, orange), $u_{3}\left(r\right)$ (third order, green), and $u_{4}\left(r\right)$ (fourth order, blue), as defined in Equation 8. The dotted line marks the Coulomb potential $l_{B}/r$ .

To capture the long-range part of the electrostatic interaction, we choose a truncated Coulomb potential

$$u\left(r\right)=\frac{l_{B}}{2r}\;\left(\frac{r}{r_{0}}+1-\left|\frac{r}{r_{0}}-1\right|\right),$$ (5)

where $l_{B}$ is the Bjerrum length and $r_{0}$ is the truncation length. Truncated Coulomb potentials have been employed in the past to cut off the long-range part with the goal to facilitate efficient computer simulations (Linse and Andersen, 1986; Baker et al., 1999). In contrast, we propose to cut off the short-range part of the Coulomb interaction, as shown in Figure 1: the black solid line in both diagrams of Figure 1 shows the truncated potential u(r) according to Equation 5. The operator A can be identified conveniently in Fourier space, where the Fourier transformation

$$\tilde{u}\left(k\right)=\frac{4\pi }{k}\;\overset{\infty }{\underset{0}{\int }}dr\;r\sin\left(kr\right)u\left(r\right)=4\pi l_{B}\frac{\sin\left(kr_{0}\right)}{k^{3}r_{0}},$$

together with $\tilde{A}\tilde{u}\left(k\right)=-1$, gives rise to

$$\begin{array}{ccc} A & = & \frac{\nabla^{2}}{4\pi l_{B}}\;\frac{r_{0}\nabla }{\sinh\left(r_{0}\nabla \right)}\\ & = & \frac{\nabla^{2}}{4\pi l_{B}}\;\overset{\infty }{\underset{i=0}{\sum }}\frac{\left(2-4^{i}\right)B_{2i}}{\left(2i\right)!}{\left(r_{0}\nabla \right)}^{2i}=\frac{\nabla^{2}}{4\pi l_{B}}\;\left[1-\frac{1}{6}{\left(r_{0}\nabla \right)}^{2}+\frac{7}{360}{\left(r_{0}\nabla \right)}^{4}\mp \cdots \;\right]. \end{array}$$ (6)

In Equation 6, ∇ and $B_{i}$ denote the nabla differential operator and the *i*th Bernoulli number, respectively. Recall that the set of Bernoulli numbers can be defined via a series expansion of the generating function $x/\left(e^{x}-1\right)=\sum_{i=0}^{\infty }B_{i}x^{i}/i!$ . If the truncated Coulomb potential is approximated by the j -th order operator,

$$A_{j}=\frac{\nabla^{2}}{4\pi l_{B}}\;\overset{j}{\underset{i=0}{\sum }}\frac{\left(2-4^{i}\right)B_{2i}}{\left(2i\right)!}{\left(r_{0}\nabla \right)}^{2i},$$ (7)

then the corresponding potential is given by the following equation:

$$u_{j}\left(r\right)=\frac{2}{\pi }\;\frac{l_{B}}{r}\overset{\infty }{\underset{0}{\int }}dk\;\frac{\sin\left(kr\right)}{k}\frac{1}{\overset{j}{\underset{i=0}{\sum }}\frac{\left(2-4^{i}\right)B_{2i}}{\left(2i\right)!}{\left(-k^{2}r_{0}^{2}\right)}^{i}}.$$ (8)

The function $u_{j}\left(r\right)$ in Equation 8 is the j -th order approximation of the truncated Coulomb potential. For j=0 , Equation 8 gives rise to the Coulomb potential $u_{0}\left(r\right)=l_{B}/r$ . In the limit j→∞ , the truncated Coulomb potential $u_{j\to \infty }\left(r\right)=u\left(r\right)$ , as specified in Equation 5, is recovered. As has been pointed out by Lee et al. (2015), the first-order approximation of the truncated Coulomb potential is as follows:

$$u_{1}\left(r\right)=\frac{l_{B}}{r}\;\left(1-e^{-\sqrt{6}\frac{r}{r_{0}}}\right).$$ (9)

The right diagram in Figure 1 shows the truncated Coulomb potential u(r) together with the increasingly more accurate approximations $u_{1}\left(r\right)$ , $u_{2}\left(r\right)$ , $u_{3}\left(r\right)$ , and $u_{4}\left(r\right)$ . In the present work, we will focus on the first-order approximation $u_{1}\left(r\right)$ only, leading to a fourth-order Poisson equation $A_{1}\Psi =-\eta /\nu$ that utilizes the differential operator $A_{1}=\nabla^{2}\left[1-{\left(r_{0}\nabla \right)}^{2}/6\right]/\left(4\pi l_{B}\right)$ similar to the BSK model (Bazant et al., 2011). However, Equations 7, 8 provide a method for the incorporation of higher-order approximations in a straightforward manner.

We point out that the BSK model (Bazant et al., 2011) is an effective mean-field approach based on the pair potential $u_{1}\left(r\right)$ . The emergence of $u_{1}\left(r\right)$ from the bare Coulomb potential can be the result of charge discreteness, which we consider in the present work, or of short-range correlations. Our present work considers electrostatic correlations beyond the short-range correlations of the BSK model. Electrostatic correlations between ion pairs are not accounted for by the BSK model.

### 2.2 Free energy minimization

Before formulating and minimizing a free energy that accounts for charge discreteness and electrostatic correlations between ion pairs, we start with three comments: first, choosing the truncation radius $r_{0}$ to be equal or slightly larger than the lattice spacing b enables us to explicitly include the electrostatic interactions between neighboring ions: $\omega_{11}=\omega_{22}=\omega =l_{B}/b$ between cation–cation and anion–anion pairs and $\omega_{12}=\omega_{21}=-\omega =-l_{B}/b$ between cation–anion and anion–cation pairs. This introduces the definition $\omega =l_{B}/b$ and the notation $\omega_{ij}$ that we use in Equation 10. Second, we account for electrostatic correlations between ions located at neighboring lattice sites through QCA (Davis, 1996). Correlations involving more than two ions can, in principle, be incorporated into QCA (Bossa et al., 2015), but they are ignored in the present work. To account for correlated ion pairs, we consider the local fraction $\phi_{ij}=\phi_{ij}\left(r\right)$ of i - j pairs, where the indices i and j adopt the value 1 for a cation and 2 for an anion. Third, we introduce the chemical potentials $\mu_{1}$ and $\mu_{2}$ of the cations and anions, respectively, and choose them to ensure coexistence with a bulk phase of the ionic liquid, where $\phi_{1}=\phi_{2}=1/2$ . We also recall the definition of the lattice coordination number z . With that, we express the free energy of a lattice-based ionic liquid as follows (Downing et al., 2018b):

$$\begin{array}{ccc} F & = & \frac{1}{\nu }\;\int d^{3}r\left[-\frac{\nu }{2}\Psi \;A\Psi +\left(1-z\right)\left(\phi_{1}\ln\phi_{1}+\phi_{2}\ln\phi_{2}\right)\right.\\ & \left.+\frac{z}{2}\overset{2}{\underset{ij=1}{\sum }}\left(\phi_{ij}\ln\phi_{ij}+\omega_{ij}\phi_{ij}\right)-\mu_{1}\phi_{1}-\mu_{2}\phi_{2}\right]. \end{array}$$ (10)

The four contributions to this free energy account for the electrostatic energy due to the long-range part of the Coulomb potential, the mixing entropy of ion pairs according to QCA, the electrostatic energy associated with neighboring ion pairs, and a Legendre transformation that fixes the chemical potentials of the cations and anions in the ionic liquid. The $\phi_{ij}$ in Equation 10 must fulfill the three conservation relations $\phi_{1}=\phi_{11}+\phi_{12}$ , $\phi_{2}=\phi_{21}+\phi_{22}$ , and $\phi_{12}=\phi_{21}$ . This leaves one degree of freedom for the local distribution of ion pairs, which we define conveniently as $\bar{\phi }=\phi_{12}+\phi_{21}$ . The difference in mole fractions $\eta =\phi_{1}-\phi_{2}$ constitutes another degree of freedom. We thus have the following equation:

$$\phi_{1}=\frac{1+\eta }{2},\phi_{2}=\frac{1-\eta }{2},\phi_{11}=\phi_{1}-\frac{\bar{\phi }}{2},\phi_{22}=\phi_{2}-\frac{\bar{\phi }}{2},\phi_{12}=\phi_{21}=\frac{\bar{\phi }}{2}.$$ (11)

Note that both $\bar{\phi }=\bar{\phi }\left(r\right)$ and η=η(r) depend on the position r inside the ionic liquid. Calculation of the first variation in the free energy $F=F\left(\eta ,\bar{\phi }\right)$ leads to

$$\begin{array}{ccc} \delta F & = & \frac{1}{\nu }\;\int d^{3}r\left\{\delta \eta \left[\Psi +\frac{1-z}{2}\ln\frac{1+\eta }{1-\eta }+\frac{z}{4}\ln\frac{1+\eta -\bar{\phi }}{1-\eta -\bar{\phi }}-\frac{1}{2}\left(\mu_{1}-\mu_{2}\right)\right]\right.\\ & + & \left.\delta \bar{\phi }\left[\frac{z}{4}\ln\frac{{\bar{\phi }}^{2}}{\left(1+\eta -\bar{\phi }\right)\;\left(1-\eta -\bar{\phi }\right)}-z\omega \right]\right\}. \end{array}$$ (12)

We have not included boundary terms in Equation 12 because they will be discussed separately below. By symmetry, the chemical potentials $\mu_{1}=\mu_{2}$ are equal. Thermal equilibrium demands δF=0 , thus leading to the following two equations:

$$e^{4\omega }=\frac{{\bar{\phi }}^{2}}{{\left(1-\bar{\phi }\right)}^{2}-\eta^{2}},0=2\Psi +\left(1-z\right)\ln\frac{1+\eta }{1-\eta }+\frac{z}{2}\ln\frac{1+\eta -\bar{\phi }}{1-\eta -\bar{\phi }},$$ (13)

which must be fulfilled at each position r . Equation 13 defines the two relations η=η(Ψ) and $\bar{\phi }=\bar{\phi }\left(\Psi \right)$, with ω and z as additional parameters. Spatial compositional variations arise from the solutions of Equation 3. However, to simplify calculations, we replace the operator A by its first-order approximation $A_{1}$, as defined in Equation 7. This renders the level of modeling the long-range part of the electrostatic interactions similar to the BSK model (Bazant et al., 2011) yet with different boundary conditions as discussed below. Equation 3 then reads as follows:

$$l^{2}\nabla^{2}\left(1-\frac{r_{0}^{2}}{6}\nabla^{2}\right)\Psi =-\eta \left(\Psi \right),$$ (14)

where we have defined the length $l=\sqrt{\nu /\left(4\pi l_{B}\right)}$ . For example, $l_{B}=1\;\text{nm}$ and $\nu =1\;{\text{nm}}^{3}$ imply l=0.3nm . We will use l as unit length throughout this work. Equation 14 is a self-consistency relationship that takes the form of a fourth-order differential equation with the function η(Ψ) defined through a set of two algebraic equations. As pointed out above, electrostatic interactions are represented by the potential $u_{1}\left(r\right)$ in Equation 9. Unlike interpretations in terms of short-range ion correlations (Storey and Bazant, 2012; Moon et al., 2015; Blossey et al., 2017; Shalabi et al., 2019; de Souza and Bazant, 2020), we associate the use of $u_{1}\left(r\right)$ with an approximation of the truncated Coulomb potential u(r) in Equation 5, which originates in our goal to model charge discreteness rather than correlations. A complementary interpretation of $u_{1}\left(r\right)$ would be in terms of excluded volume interactions between adjacent ions, and such an interpretation has been proposed recently (Gupta et al., 2020a).

**FIGURE 2:**
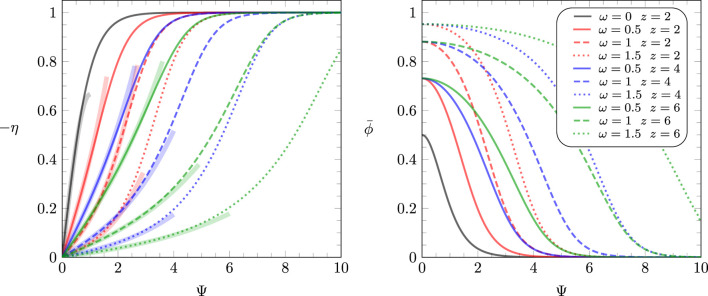
Relationships η=η(Ψ) (left diagram) and $\bar{\phi }\left(\Psi \right)$ (right diagram) according to Equation 13. Curves of the same color refer to z=2 (red), z=4 (blue), and z=6 (green) and are calculated for ω=1/2 (solid colored lines), ω=1 (broken lines), and ω=1.5 (dotted lines). For ω=0 , Equation 13 predicts η=−tanh(Ψ) irrespective of z (the black curve on the left diagram). All curves colored red (z=2) follow the explicit expressions in Equation 15. The thick transparent lines in the left diagram represent third-order approximations of η(Ψ) , as specified in Equations 29, 30. They will be utilized in Sections 3.1, 3.2 to calculate the differential capacitance for the linearized model and beyond that using a perturbation approach.

The function η=η(Ψ) that is needed to solve Equation 14 satisfies Equation 13 and is displayed together with $\bar{\phi }\left(\Psi \right)$ in Figure 2 for different parameters z and ω . The solution of Equation 13 for ω=0 is η=−tanh(Ψ) , irrespective of z , and is shown as a black line in the left diagram of Figure 2. Curves of the same color refer to z=2 (red), z=4 (blue), and z=6 (green) and are calculated for different values of ω , as indicated in the legend. Note that for z=2 (the red lines in Figure 2), Equation 13 yields the following simple analytic result:

$$\eta \left(\Psi \right)=-\frac{\sinh\Psi }{\sqrt{e^{4\omega }+\sinh^{2}\Psi }},\bar{\phi }\left(\Psi \right)=\frac{e^{2\omega }-\sqrt{1+\left(e^{4\omega }-1\right)\eta {\left(\Psi \right)}^{2}}}{2\sinh\left(2\omega \right)},$$ (15)

which corresponds to the solution of the one-dimensional Ising model (Davis, 1996) in an external field Ψ .

### 2.3 Boundary conditions

From this point forward, we consider a single charged planar electrode that carries a uniform surface charge density σ and is in contact with the ionic liquid. We locate the origin of a Cartesian coordinate system at the electrode surface, with its x -axis pointing normal into the ionic liquid. The electrostatic potential Ψ=Ψ(x) then depends only on the distance x to the electrode, and Equation 14 reads as follows:

$$l^{2}\Psi^{\prime \prime }\left(x\right)-l^{2}\frac{r_{0}^{2}}{6}\Psi \prime \prime \prime \prime \left(x\right)=-\eta \left(\Psi \left(x\right)\right),$$ (16)

where a prime denotes the derivative with respect to the position x . We derive solutions of this fourth-order non-linear differential equation for x≥0 subject to the boundary conditions $\Psi \left(x\to \infty \right)=\Psi^{\prime }\left(x\to \infty \right)=0$ as well as

$$\Psi^{\prime }\left(0\right)=0,\;\Psi \prime \prime \prime \left(0\right)=\frac{6s}{l\;r_{0}^{2}},$$ (17)

where $s=\nu \sigma /\left(le\right)=4\pi l_{B}l\sigma /e$ is a scaled (dimensionless) surface charge density. Different sets of boundary conditions at the electrode surface x=0 have been proposed in the past for fourth-order differential equations of the type in Equation 16: $\Psi^{\prime }\left(0\right)=-s/l$ and Ψ‴(0)=0 in the original BSK model (Bazant et al., 2011) and $\Psi^{\prime }\left(0\right)=-s/l$ and $\Psi^{\prime \prime }\left(0\right)=0$ based on variational free energy minimization (Gupta et al., 2020b). In addition, $\Psi^{\prime }\left(0\right)=-s/l$ and $\left(r_{0}/\sqrt{6}\right)\times \Psi \prime \prime \prime \left(0\right)=\Psi^{\prime \prime }\left(0\right)$ have been proposed based on the continuity of the Maxwell stress tensor at the charged electrode (de Souza and Bazant, 2020) and on interpreting $u_{1}\left(r\right)$ in Equation 9 as a specific combination of Coulomb and Yukawa potentials (Bossa and May, 2020). We take the point of view that the truncated Coulomb potential applies to all charges in the system, including those within the ionic liquid and those at the electrode surface. In this case, the energy (per unit area A ) of the system associated with the long-range part of the electrostatic interactions is as follows:

$$\begin{array}{cccc} \frac{U}{A} & = & \frac{l^{2}}{2\nu }\overset{\infty }{\underset{0}{\int }}dx\;\left({\Psi^{\prime }}^{2}+\frac{r_{0}^{2}}{6}{\Psi^{\prime \prime }}^{2}\right) & =-\frac{l^{2}}{2\nu }\left.\{,\Psi \left(0\right)\left(\Psi^{\prime }\left(0\right)-\frac{r_{0}^{2}}{6}\Psi \prime \prime \prime \left(0\right)\right)+\frac{r_{0}^{2}}{6}\Psi^{\prime }\left(0\right)\Psi^{\prime \prime }\left(0\right)+\overset{\infty }{\underset{0}{\int }}dx\;\Psi \left(\Psi^{\prime \prime }-\frac{r_{0}^{2}}{6}\Psi \prime \prime \prime \prime \right)\right\}\}. \end{array}$$ (18)

Its first variation is as follows:

$$\frac{\delta U}{A}=-\frac{l^{2}}{\nu }\left.\{,\Psi \left(0\right)\left(\delta \Psi^{\prime }\left(0\right)-\frac{r_{0}^{2}}{6}\delta \Psi \prime \prime \prime \left(0\right)\right)+\frac{r_{0}^{2}}{6}\Psi^{\prime }\left(0\right)\delta \Psi^{\prime \prime }\left(0\right)+\overset{\infty }{\underset{0}{\int }}dx\;\Psi \left(\delta \Psi^{\prime \prime }-\frac{r_{0}^{2}}{6}\delta \Psi \prime \prime \prime \prime \right)\right\}\}.$$ (19)

The integral in Equation 19 is $\left(1/\nu \right)\;\int_{0}^{\infty }dx\;\Psi \;\delta \eta$, which, when combined with the variation in the non-electrostatic contributions to the free energy, will vanish, as demonstrated in Equation 12. Poisson’s equation for the truncated Coulomb potential, $l^{2}/\nu \left[\Psi^{\prime \prime }-\left(r_{0}^{2}/6\right)\Psi \prime \prime \prime \prime \right]=-\eta /\nu$ , relates the potential to the local volume charge density eη/ν . Integrating that equation across the surface of the electrode yields $l\left[\Psi^{\prime }\left(0\right)-\left(r_{0}^{2}/6\right)\Psi \prime \prime \prime \left(0\right)\right]=-s$ . Because the scaled surface charge density s is fixed, the ensuing relation $\delta \Psi^{\prime }\left(0\right)-\left(r_{0}^{2}/6\right)\times \delta \Psi \prime \prime \prime \left(0\right)=0$ causes the first surface term in Equation 19 to vanish. Vanishing of the second surface term demands $\Psi^{\prime }\left(0\right)=0$ . Because of $\Psi^{\prime }\left(0\right)=0$ , the term $\left(r_{0}^{2}/6\right)\times \Psi^{\prime }\left(0\right)\Psi^{\prime \prime }\left(0\right)$ in the third line of Equation 18 vanishes. The remaining two terms, the electrostatic energy due to the surface charges on the electrode and the electrostatic energy due the ions in the ionic liquid, then render Equation 18 identical to Equation 4. Hence, the boundary conditions at the electrode, $\Psi^{\prime }\left(0\right)=0$ and $\Psi \prime \prime \prime \left(0\right)=6s/\left(lr_{0}^{2}\right)$ , lead to the consistent vanishing of δF with F defined in Equation 10, including all surface terms.

The following three comments complete our discussion of the boundary conditions. First, for x<0, the potential is constant, Ψ(x)=Ψ(0) . Hence, because the region x<0 does not contribute to U , the integration in Equation 18 may start from x=0 instead of also including negative values of x . Second, short-range electrostatic interactions of the ionic liquid with the electrode surface contribute another surface term −η(0)sl/b to U/A in Equation 18. Because the variation of this term vanishes, it does not add to the boundary conditions. Third, our approach recovers the boundary condition $\Psi^{\prime }\left(0\right)=-s/l$ , used previously to solve the second-order self-consistency differential equation $l^{2}\Psi^{\prime \prime }\left(x\right)=-\eta \left(\Psi \left(x\right)\right)$ that results from our model for $r_{0}=0$ (Downing et al., 2018b).

The solution Ψ(x) of Equation 16 reveals how the surface potential $\Psi_{0}=\Psi \left(x=0\right)$ depends on the scaled surface charge density s , allowing us to compute the differential capacitance as follows:

$$C_{\mathrm{diff}}=\frac{\epsilon \epsilon_{0}}{l}\frac{ds}{d\Psi_{0}}=\frac{\epsilon \epsilon_{0}}{l}{\bar{C}}_{\mathrm{diff}}.$$

Calculation of the scaled differential capacitance ${\bar{C}}_{\mathrm{diff}}=ds/d\Psi_{0}$ is a major focus of the present work.

## 3 Results and discussion

Before computing general results for ${\bar{C}}_{\mathrm{diff}}\left(s\right)$ numerically, we analytically derive quadratic expressions of the form ${\bar{C}}_{\mathrm{diff}}={\bar{C}}_{\mathrm{diff}}^{\mathrm{lin}}+s^{2}\times \Omega /2$ through linearizing our model followed by a perturbation approach to access the nonlinear regime. The determination of the two constants ${\bar{C}}_{\mathrm{diff}}^{\mathrm{lin}}$ and Ω as functions of $r_{0}$ , ω , and z provides insights about the role of charge discreteness and electrostatic correlations between ion pairs.

### 3.1 Linearized theory

For small potentials, Ψ≪1 , only the linear part of the relationship η(Ψ) is significant. We use Equation 13 to perform a series expansion of η(Ψ) up to the linear order. The result, $\eta =-\Psi /\left[1+\left(z/2\right)\times \left(e^{2\omega }-1\right)\right]$ , can conveniently be expressed as $\eta =-\left(3/2\right)\times {\left(l/r_{c}\right)}^{2}\times \Psi$ , where we have defined the length:

$$r_{c}=l\sqrt{\frac{3}{2}\left[1+\frac{z}{2}\left(e^{2\omega }-1\right)\right]}.$$ (20)

This length will be shown to play a role in separating different regimes of the solution Ψ(x) for the linear problem. We thus solve the equation

$$\Psi^{\prime \prime }\left(x\right)-\frac{r_{0}^{2}}{6}\Psi \prime \prime \prime \prime \left(x\right)=\frac{3}{2r_{c}^{2}}\Psi \left(x\right),$$

subject to $\Psi^{\prime }\left(0\right)=0$ , $\Psi \prime \prime \prime \left(0\right)=\left(6/r_{0}^{2}\right)\times \left(s/l\right)$ , $\Psi \left(x\to \infty \right)=\Psi^{\prime }\left(x\to \infty \right)=0$ . The solution of the linear problem, denoted in the following by $\Psi_{\mathrm{lin}}\left(x\right)$ , can generally be expressed as the sum of two contributions:

$$\Psi_{\mathrm{lin}}\left(x\right)=s\left[a_{1}e^{-\lambda_{1}\frac{x}{r_{0}}}+a_{2}e^{-\lambda_{2}\frac{x}{r_{0}}}\right],$$ (21)

with

$$\frac{\lambda_{1}}{\sqrt{3}}=\sqrt{1+\sqrt{1-{\left(\frac{r_{0}}{r_{c}}\right)}^{2}}},\frac{\lambda_{2}}{\sqrt{3}}=\sqrt{1-\sqrt{1-{\left(\frac{r_{0}}{r_{c}}\right)}^{2}}}.$$ (22)

The two constants $a_{1}$ and $a_{2}$ follow from the boundary conditions in Equation 17,

$$a_{1}=-\frac{r_{0}}{\sqrt{3}\;l}\;\frac{1}{\sqrt{1-{\left(\frac{r_{0}}{r_{c}}\right)}^{2}}\sqrt{1+\sqrt{1-{\left(\frac{r_{0}}{r_{c}}\right)}^{2}}}},a_{2}=\frac{r_{0}}{\sqrt{3}\;l}\;\frac{1}{\sqrt{1-{\left(\frac{r_{0}}{r_{c}}\right)}^{2}}\sqrt{1-\sqrt{1-{\left(\frac{r_{0}}{r_{c}}\right)}^{2}}}}.$$ (23)

In the following, we discuss how the structure of the solution changes as $r_{0}$ is varied. For $r_{0}=0$ , the corresponding potential,

$$\Psi_{\mathrm{lin}}\left(x\right)=\sqrt{\frac{2}{3}}\;\frac{r_{c}}{l}\;s\;e^{-\left(\sqrt{\frac{3}{2}}\;\frac{x}{r_{c}}\right)},$$ (24)

is characterized by a single decay length. For $0<r_{0}<r_{c}$ , there is a double-exponential decay with the two decay lengths $r_{0}/\lambda_{1}$ and $r_{0}/\lambda_{2}$ . At $r_{0}=r_{c}$ , the decay length $r_{0}/\lambda_{2}$ diverges, and the potential becomes as follows:

$$\Psi_{\mathrm{lin}}\left(x\right)=s\;\frac{r_{c}}{l}\;\left(\frac{1}{\sqrt{3}}+\frac{x}{r_{c}}\right)\;e^{-\left(\sqrt{3}\;\frac{x}{r_{c}}\right)}.$$ (25)

Finally, for $r_{0}>r_{c}$ , the quantities $\lambda_{1}=\lambda_{3}+i\lambda_{4}$ and $\lambda_{2}=\lambda_{3}-i\lambda_{4}$ , as well as $a_{1}=a_{3}-ia_{4}$ and $a_{2}=a_{3}+ia_{4}$ , adopt complex conjugate values with

$$\lambda_{3}=\sqrt{\frac{3}{2}\left(\frac{r_{0}}{r_{c}}+1\right)},\;\lambda_{4}=\sqrt{\frac{3}{2}\left(\frac{r_{0}}{r_{c}}-1\right)}$$

and

$$a_{3}=\frac{r_{c}}{\sqrt{6}l}\;\frac{1}{\sqrt{\frac{r_{0}}{r_{c}}+1}},\;a_{4}=\frac{r_{c}}{\sqrt{6}l}\;\frac{1}{\sqrt{\frac{r_{0}}{r_{c}}-1}}.$$

**FIGURE 3:**
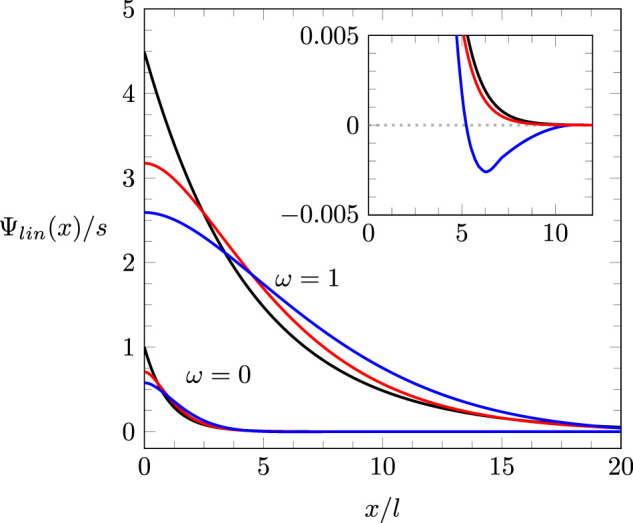
$\Psi_{\mathrm{lin}}\left(x\right)/s$ for ω=1 (upper three curves) and ω=0 (lower three curves, magnified in the inset), with $r_{0}=0$ (black), $r_{0}=r_{c}$ (red), and $r_{0}=2r_{c}$ (blue). All curves are calculated for z=6 . The black curves $\left(r_{0}=0\right)$ are single exponentials according to Equation 24 with a decay length l for ω=0 and 4.5×l for ω=1 . The red curves $\left(r_{0}=r_{c}\right)$ are specified by Equation 25. The blue curves, which follow Equation 26, exhibit exponentially decaying oscillations.

The potential

$$\Psi_{\mathrm{lin}}\left(x\right)=2s\;e^{-\left(\frac{\lambda_{3}x}{r_{0}}\right)}\left[a_{3}\cos\left(\frac{\lambda_{4}x}{r_{0}}\right)+a_{4}\sin\left(\frac{\lambda_{4}x}{r_{0}}\right)\right]$$ (26)

then exhibits exponentially decaying oscillations. Figure 3 shows $\Psi_{\mathrm{lin}}\left(x\right)/s$ for ω=1 (upper three curves) and ω=0 (lower three curves) and three different ratios of $r_{0}/r_{c}$ , namely $r_{0}=0$ (black), $r_{0}=r_{c}$ (red), and $r_{0}=2r_{c}$ (blue). Figure 3 suggests the tendency of ω to increase the magnitude and decay length of the potential. The truncation length $r_{0}$ only moderately modifies this tendency. Irrespective of the ratio $r_{0}/r_{c}$ , the same linear relationship,

$$\Psi_{0}^{\mathrm{lin}}=s\;\sqrt{\frac{2}{3}}\;\frac{r_{c}}{l}\;\frac{1}{\sqrt{1+\frac{r_{0}}{r_{c}}}},$$ (27)

between surface potential $\Psi_{0}^{\mathrm{lin}}=\Psi_{\mathrm{lin}}\left(x=0\right)$ and scaled surface charge density s results. Hence, Equation 27 is valid within the linearized model for any choice of z , ω , and $r_{0}$ . This leads to a prediction for the differential capacitance ${\bar{C}}_{\mathrm{diff}}\left(s=0\right)={\bar{C}}_{\mathrm{diff}}^{\mathrm{lin}}$ in the limit of an uncharged electrode, where both s=0 and $\Psi_{0}=0$ ,

$${\bar{C}}_{\mathrm{diff}}^{\mathrm{lin}}=\frac{ds}{d\Psi_{0}^{\mathrm{lin}}}=\frac{\sqrt{1+\frac{r_{0}}{r_{c}}}}{\sqrt{1+\frac{z}{2}\left(e^{2\omega }-1\right)}}.$$ (28)

Recall that $r_{c}$ depends on z and ω through Equation 20. Clearly, increasing ω lowers ${\bar{C}}_{\mathrm{diff}}^{\mathrm{lin}}$ , whereas larger $r_{0}$ increases ${\bar{C}}_{\mathrm{diff}}^{\mathrm{lin}}$ .

### 3.2 Non-linear model: a perturbation approach

The linearized model is based on the relationship η∼Ψ . In this section, we employ a perturbation approach to analytically model the nonlinear region, where the potential deviates only slightly from $\Psi_{\mathrm{lin}}$ . To this end, we expand the relationship η(Ψ) up to the third order,

$$-\eta =\frac{3}{2}\;{\left(\frac{l}{r_{c}}\right)}^{2}\Psi +b_{3}\Psi^{3}.$$ (29)

From Equation 13, we find the third-order coefficient as follows:

$$b_{3}=\frac{\frac{z}{2}\left(e^{2\omega }+2\right){\left(e^{2\omega }-1\right)}^{2}-2}{6{\left[1+\frac{z}{2}\left(e^{2\omega }-1\right)\right]}^{4}}.$$ (30)

The left diagram in Figure 2 displays the third-order approximation, as specified in Equations 29, 30 (color-matching thick transparent lines). Hence, we aim to solve the nonlinear differential equation

$$\Psi^{\prime \prime }\left(x\right)-\frac{r_{0}^{2}}{6}\Psi \prime \prime \prime \prime \left(x\right)=\frac{3}{2r_{c}^{2}}\Psi \left(x\right)+\frac{b_{3}}{l^{2}}\Psi^{3}\left(x\right),$$ (31)

subject to $\Psi^{\prime }\left(0\right)=0$ , $\Psi \prime \prime \prime \left(0\right)=\left(6/r_{0}^{2}\right)\times \left(s/l\right)$ , and $\Psi \left(x\to \infty \right)=\Psi^{\prime }\left(x\to \infty \right)=0$ . We express the potential $\Psi \left(x\right)=\Psi_{\mathrm{lin}}\left(x\right)+b_{3}\Psi_{\mathrm{per}}\left(x\right)$ as the sum of the solution for the linear problem $\left(\Psi_{\mathrm{lin}}\right)$ and a small perturbation $\left(b_{3}\Psi_{\mathrm{per}}\right)$ . Substituting Ψ(x) into Equation 31 leads to the linear, inhomogeneous differential equation

$$\Psi_{per}^{\prime \prime }\left(x\right)-\frac{r_{0}^{2}}{6}\Psi \prime \prime {\prime \prime }_{per}\left(x\right)=\frac{3}{2r_{c}^{2}}\Psi_{\mathrm{per}}\left(x\right)+\frac{1}{l^{2}}\Psi_{\mathrm{lin}}^{3}\left(x\right)$$ (32)

for the perturbation contribution of the potential $\Psi_{\mathrm{per}}\left(x\right)$ , where $\Psi_{\mathrm{lin}}\left(x\right)$ is given by Equation 21. The solution $\Psi_{\mathrm{per}}\left(x\right)=\Psi_{\mathrm{inh}}\left(x\right)+\Psi_{\mathrm{hom}}\left(x\right)$ of Equation 32 can be expressed as the sum of a specific solution for the inhomogeneous equation (index “inh”):

$$\begin{array}{ccc} \Psi_{\mathrm{inh}}\left(x\right) & = & s^{3}{\left(\frac{r_{0}}{l}\right)}^{2}\left[\frac{a_{1}^{3}e^{-3\lambda_{1}\frac{x}{r_{0}}}}{{\left(3\lambda_{1}\right)}^{2}-\frac{1}{6}{\left(3\lambda_{1}\right)}^{4}-\frac{3}{2}{\left(\frac{r_{0}}{r_{c}}\right)}^{2}}+\frac{3a_{1}^{2}a_{2}e^{-\left(2\lambda_{1}+\lambda_{2}\right)\frac{x}{r_{0}}}}{{\left(2\lambda_{1}+\lambda_{2}\right)}^{2}-\frac{1}{6}{\left(2\lambda_{1}+\lambda_{2}\right)}^{4}-\frac{3}{2}{\left(\frac{r_{0}}{r_{c}}\right)}^{2}}\right.\\ & \left.+\frac{3a_{1}a_{2}^{2}e^{-\left(\lambda_{1}+2\lambda_{2}\right)\frac{x}{r_{0}}}}{{\left(\lambda_{1}+2\lambda_{2}\right)}^{2}-\frac{1}{6}{\left(\lambda_{1}+2\lambda_{2}\right)}^{4}-\frac{3}{2}{\left(\frac{r_{0}}{r_{c}}\right)}^{2}}+\frac{a_{2}^{3}e^{-3\lambda_{2}\frac{x}{r_{0}}}}{{\left(3\lambda_{2}\right)}^{2}-\frac{1}{6}{\left(3\lambda_{2}\right)}^{4}-\frac{3}{2}{\left(\frac{r_{0}}{r_{c}}\right)}^{2}}\right], \end{array}$$

where $\lambda_{1}$ , $\lambda_{2}$ , $a_{1}$, and $a_{2}$ are defined in Equations 22, 23, and a solution for the homogeneous equation (index “hom”):

$$\Psi_{\mathrm{hom}}\left(x\right)=s^{3}\left[c_{1}e^{-\lambda_{1}\frac{x}{r_{0}}}+c_{2}e^{-\lambda_{2}\frac{x}{r_{0}}}\right].$$ (33)

The two constants $c_{1}$ and $c_{2}$ in Equation 33 can be determined such that besides $\Psi_{\mathrm{per}}\left(x\to \infty \right)=\Psi_{\mathrm{per}}^{\prime }\left(x\to \infty \right)=0$ , Equation 32 also satisfies the boundary conditions $\Psi_{\mathrm{per}}^{\prime }\left(0\right)=0$ and $\Psi_{\mathrm{per}}^{\prime \prime \prime }\left(0\right)=0$ . This results in an expression for the surface contribution of the perturbation potential $\Psi_{\mathrm{per}}\left(0\right)=s^{3}B$ with

$$B=-\frac{1}{4}\;{\left[1+\frac{z}{2}\left(e^{2\omega }-1\right)\right]}^{\frac{5}{2}}\times \frac{1+\frac{25}{6}\frac{r_{0}}{r_{c}}+\frac{41}{12}{\left(\frac{r_{0}}{r_{c}}\right)}^{2}}{{\left(1+\frac{r_{0}}{r_{c}}\right)}^{\frac{5}{2}}\left(1+\frac{5}{3}\frac{r_{0}}{r_{c}}\right)}.$$ (34)

The total surface potential $\Psi_{0}=\Psi_{\mathrm{lin}}\left(0\right)+b_{3}\Psi_{\mathrm{per}}\left(0\right)=s/{\bar{C}}_{\mathrm{diff}}^{\mathrm{lin}}+s^{3}Bb_{3}$ can be used to calculate the scaled differential capacitance,

$${\bar{C}}_{\mathrm{diff}}=\frac{1}{\frac{d\Psi_{0}}{ds}}={\bar{C}}_{\mathrm{diff}}^{\mathrm{lin}}-3Bb_{3}{\left({\bar{C}}_{\mathrm{diff}}^{\mathrm{lin}}\right)}^{2}s^{2}={\bar{C}}_{\mathrm{diff}}^{\mathrm{lin}}+\frac{\Omega }{2}s^{2},$$ (35)

analytically up to quadratic order in s . Using the expressions for ${\bar{C}}_{\mathrm{diff}}^{\mathrm{lin}}$ , $b_{3}$ , and B in Equations 28, 30, 34, respectively, to calculate the quadratic-order coefficient $\Omega =-6Bb_{3}{\left({\bar{C}}_{\mathrm{diff}}^{\mathrm{lin}}\right)}^{2}$ yields the following:

$$\Omega =\frac{\frac{z}{2}\left(e^{2\omega }+2\right){\left(e^{2\omega }-1\right)}^{2}-2}{4{\left[1+\frac{z}{2}\left(e^{2\omega }-1\right)\right]}^{\frac{5}{2}}}\times \frac{1+\frac{25}{6}\frac{r_{0}}{r_{c}}+\frac{41}{12}{\left(\frac{r_{0}}{r_{c}}\right)}^{2}}{{\left(1+\frac{r_{0}}{r_{c}}\right)}^{\frac{3}{2}}\left(1+\frac{5}{3}\frac{r_{0}}{r_{c}}\right)}.$$ (36)

The quadratic expression for ${\bar{C}}_{\mathrm{diff}}\left(s\right)$ in Equation 35 with the analytic results for ${\bar{C}}_{\mathrm{diff}}^{\mathrm{lin}}$ and Ω given in Equations 28, 36 is the central outcome of this work, accounting (albeit on an approximate level) for both charge discreteness and electrostatic correlations.

### 3.3 Limiting cases

#### 3.3.1 Continuum mean-field model, $r_{0}=0$ and ω=0

For $r_{0}=0$ and ω=0 , Equation 16 reduces to the well-known classical mean-field equation $l^{2}\Psi^{\prime \prime }\left(x\right)=\tanh\Psi$ that emerges from the lattice-based Poisson–Boltzmann framework of a solvent-free ionic liquid (Borukhov et al., 1997; Kornyshev, 2007). Neither charge discreteness nor ion correlations are accounted for. Subject to the boundary conditions $\Psi^{\prime }\left(0\right)=-s/l$ and Ψ(x→∞)=0 , we obtain a surface potential $\Psi_{0}=arcosh\left(e^{s^{2}/2}\right)$ for x≥0 and $\Psi_{0}=-arcosh\left(e^{s^{2}/2}\right)$ for x≤0 , and thus, a scaled differential capacitance ${\bar{C}}_{\mathrm{diff}}={\left(d\Psi_{0}/ds\right)}^{-1}=\sqrt{1-e^{-s^{2}}}/|s|$ . For small |s| , this can be represented by ${\bar{C}}_{\mathrm{diff}}={\bar{C}}_{\mathrm{diff}}^{\mathrm{lin}}+s^{2}\Omega /2$ with ${\bar{C}}_{\mathrm{diff}}^{\mathrm{lin}}=1$ and Ω=−1/2 . All models discussed in the present work recover this limit if both $r_{0}=0$ and ω=0 .

#### 3.3.2 Vanishing truncation length, $r_{0}=0$

For $r_{0}=0$ , the expressions for ${\bar{C}}_{\mathrm{diff}}^{\mathrm{lin}}$ and Ω in Equations 28, 36 simplify to the following:

$${\bar{C}}_{\mathrm{diff}}^{\mathrm{lin}}=\frac{1}{\sqrt{1+\frac{z}{2}\left(e^{2\omega }-1\right)}},\;\Omega =\frac{1}{4}\times \frac{\frac{z}{2}\left(e^{2\omega }+2\right){\left(e^{2\omega }-1\right)}^{2}-2}{{\left[1+\frac{z}{2}\left(e^{2\omega }-1\right)\right]}^{5/2}}.$$

This is identical to the results derived previously by Downing et al. (2018b), where an approach analogous to the present one yet without truncating the Coulomb potential was proposed. Not truncating the Coulomb potential while still adding nearest-neighbor electrostatic interactions separately into the model implies a double counting of nearest neighbor ion–ion interactions. The truncation of the Coulomb potential introduced in the present work eliminates this inconsistency.

As pointed out by Downing et al. (2018b), Ω changes sign upon increasing ω , indicating a transition from bell shape to camel shape of the differential capacitance ${\bar{C}}_{\mathrm{diff}}$ . This transition is caused by electrostatic correlations between ion pairs, as shown below by comparing the predictions for ${\bar{C}}_{\mathrm{diff}}$ in the presence and absence of electrostatic correlations.

#### 3.3.3 Vanishing central charge, ω=0

The choice ω=0 eliminates the explicit account of electrostatic interactions between nearest neighbors. Without these interactions, ion correlations between neighboring ions are no longer present. The solution of Equation 13 is $\bar{\phi }=2\phi_{1}\phi_{2}$, and the corresponding relation η=−tanhΨ implies mean-field electrostatics subject to a truncated Coulomb potential. The resulting fourth-order differential equation $l^{2}\Psi^{\prime \prime }\left(x\right)-l^{2}r_{0}^{2}\Psi \prime \prime \prime \prime \left(x\right)/6=\tanh\Psi \left(x\right)$ is equivalent to the modified Poisson–Boltzmann equation of the solvent-free BSK model (Bazant et al., 2011) yet with different boundary conditions. For ω=0, we obtain the following from Equations 28, 36:

$${\bar{C}}_{\mathrm{diff}}^{\mathrm{lin}}=\sqrt{1+\sqrt{\frac{2}{3}}\;\frac{r_{0}}{l}},\;\Omega =-\frac{1}{2}\times \frac{1+\frac{25}{6}\sqrt{\frac{2}{3}}\;\frac{r_{0}}{l}+\frac{41}{12}{\left(\sqrt{\frac{2}{3}}\;\frac{r_{0}}{l}\right)}^{2}}{{\left(1+\sqrt{\frac{2}{3}}\;\frac{r_{0}}{l}\right)}^{\frac{3}{2}}\left(1+\frac{5}{3}\sqrt{\frac{2}{3}}\;\frac{r_{0}}{l}\right)}.$$ (37)

The negative sign of Ω signifies a bell shape of the differential capacitance ${\bar{C}}_{\mathrm{diff}}\left(s\right)$ . Hence, for ω=0 , ${\bar{C}}_{\mathrm{diff}}\left(s\right)$ cannot exhibit a camel shape.

#### 3.3.4 The influence of electrostatic correlations between ion pairs

The usage of the QCA approximation accounts for electrostatic correlations between neighboring ion pairs, whereas three-body and higher-order correlations are neglected. To investigate the role of electrostatic ion pair correlations, we compare QCA to a mean-field model, which is based on a random mixing approximation (RMA) (Davis, 1996). RMA ignores ion pair correlations by modeling the entropy contribution of the free energy through an ideal lattice gas. This is accomplished by imposing $\bar{\phi }=2\phi_{1}\phi_{2}$ , which, when used in Equation 11, leads to the free energy

$$F=\frac{1}{\nu }\;\int d^{3}r\left[-\frac{\nu }{2}\Psi \;A\Psi +\phi_{1}\ln\phi_{1}+\phi_{2}\ln\phi_{2}+\omega {\left(\phi_{1}-\phi_{2}\right)}^{2}-\mu_{1}\phi_{1}-\mu_{2}\phi_{2}\right].$$

Electrostatic interactions between neighboring ions are thus described on the level of the familiar Bragg–Williams model (Davis, 1996), whereas the long-range components are accounted for by the truncated Coulomb potential. As above, we use $\phi_{1}=\left(1+\eta \right)/2$ and $\phi_{2}=\left(1-\eta \right)/2$ and note that $\mu_{1}=\mu_{2}$ . Vanishing first variation of the free energy

$$\delta F=\frac{1}{\nu }\;\int d^{3}r\delta \eta \left[\Psi -arctanh\left(\eta \right)-2\omega \eta \right]$$

then implies the relation

$$\Psi =-arctanh\left(\eta \right)-2\omega \eta .$$ (38)

This implicitly defines the function η(Ψ) to be used in the fourth-order self-consistency differential equation, Equation 14, namely, $l^{2}\Psi^{\prime \prime }\left(x\right)-l^{2}r_{0}^{2}\Psi \prime \prime \prime \prime \left(x\right)/6=-\eta \left(\Psi \left(x\right)\right)$ . Up to the third order in Ψ, this function is given by $\eta =-\Psi /\left(1+2\omega \right)+\Psi^{3}/\left[3{\left(1+2\omega \right)}^{4}\right]$ . As above, we solve the linearized model and perform a perturbation approach, yielding again an analytic expression for the scaled differential capacitance ${\bar{C}}_{\mathrm{diff}}={\bar{C}}_{\mathrm{diff}}^{\mathrm{lin}}+s^{2}\Omega /2$ up to quadratic order in the scaled surface charge density s . We find that

$${\bar{C}}_{\mathrm{diff}}^{\mathrm{lin}}=\frac{\sqrt{1+\frac{r_{0}}{{\bar{r}}_{c}}}}{\sqrt{1+2\omega }},\;\Omega =-\frac{1}{2{\left(1+2\omega \right)}^{5/2}}\;\frac{1+\frac{25}{6}\frac{r_{0}}{{\bar{r}}_{c}}+\frac{41}{12}{\left(\frac{r_{0}}{{\bar{r}}_{c}}\right)}^{2}}{{\left(1+\frac{r_{0}}{{\bar{r}}_{c}}\right)}^{3/2}\left(1+\frac{5}{3}\frac{r_{0}}{{\bar{r}}_{c}}\right)},$$ (39)

where we have defined ${\bar{r}}_{c}=l\sqrt{3\left(1+2\omega \right)/2}$ . Equation 39 accounts for the discrete nature of the ionic charges, but neglects electrostatic correlations between ion pairs. As for Equation 37, the negative sign of Ω implies a bell shape of ${\bar{C}}_{\mathrm{diff}}\left(s\right)$ . Hence, in the absence of electrostatic correlations between ion pairs, our model does not predict a transition to a camel shape of ${\bar{C}}_{\mathrm{diff}}\left(s\right)$ .

### 3.4 Numerical results

In this section, we present numerical results for ${\bar{C}}_{\mathrm{diff}}\left(s\right)$ , with various parameter choices for ω , $r_{0}$ , and z, and then contrast the predictions of the model in the presence and absence of electrostatic ion–ion correlations. Solutions of Equation 14 employ the function η(Ψ) according to Equation 13 in the presence of electrostatic ion correlations (modeled according to QCA) and according to Equation 38 in the absence of electrostatic ion correlations (modeled according to RMA).

**FIGURE 4:**
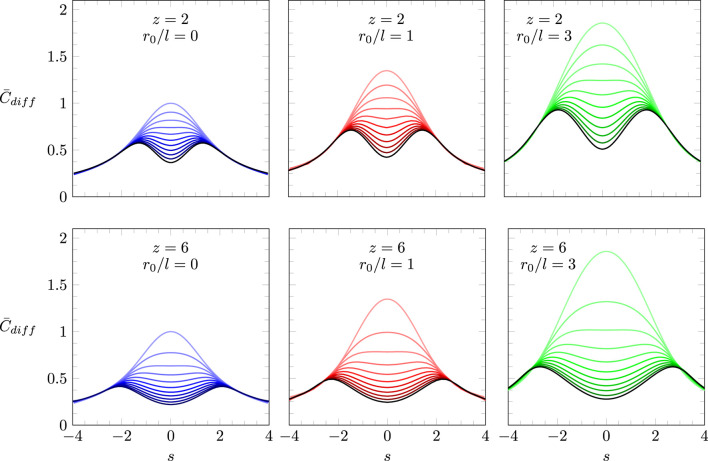
Scaled differential capacitance ${\bar{C}}_{\mathrm{diff}}$ as a function of the scaled surface charge density s based on solving Equations 13, 16, 17. Each diagram shows 11 curves for ω=0 (uppermost curve) to ω=1 (black curve on the bottom), with ω changing in steps of 0.1. Diagrams refer to z=2 (upper three diagrams) and z=6 (lower three diagrams), as well as $r_{0}=0$ (left column, colored purple), $r_{0}=l$ (middle column, colored red), and $r_{0}=3l$ (right column, colored green).

Figure 4 summarizes the predictions of our model according to Equation 16 with η(Ψ) specified in Equation 13 and the boundary conditions in Equation 17. Recall that this model incorporates both the truncated Coulomb potential and QCA, thus accounting, approximatively, for both charge discreteness and electrostatic correlations. The scaled differential capacitance ${\bar{C}}_{\mathrm{diff}}$ is displayed as a function of the scaled surface charge density s with z=2 (upper three diagrams) and z=6 (lower three diagrams), as well as $r_{0}=0$ (left, colored purple), $r_{0}=l$ (middle, colored red), and $r_{0}=3l$ (right, colored green). Each graph contains 11 curves, varying from ω=0 (the light colored curve on the top of each diagram) to ω=1 (the black curve on the bottom of each diagram) in steps of 0.1.

We point out that in the vicinity of s=0 , where ${\bar{C}}_{\mathrm{diff}}\left(s\right)={\bar{C}}_{\mathrm{diff}}^{\mathrm{lin}}+s^{2}\Omega /2$ can be represented by a quadratic function, each curve reproduces our analytic expression, as specified in Equations 28, 36. Hence, Figure 4 reinforces the validity of our analytic results and provides an extension of ${\bar{C}}_{\mathrm{diff}}\left(s\right)$ beyond the quadratic regime. As already predicted analytically, we observe the transition from a bell-shaped to a camel-shaped profile of the differential capacitance as the strength of the electrostatic interaction $\omega =l_{B}/b$ is increased. The transition occurs at a value of ω that satisfies the equation $2-3e^{2\omega }+e^{6\omega }=4/z$ . For z=2 and z=6, this amounts to ω=1/4×ln⁡3≈0.275 and ω=0.182 , respectively, independently of $r_{0}$ . The truncation length $r_{0}$ does affect the value of ${\bar{C}}_{\mathrm{diff}}\left(s=0\right)$ where the bell-to-camel shape transition occurs, but the value of ω where the transition happens is independent of $r_{0}$ . The role of $r_{0}$ is to amplify the magnitudes of ${\bar{C}}_{\mathrm{diff}}$ . This is most clearly evidenced by the explicit relationship for the dependence of ${\bar{C}}_{\mathrm{diff}}^{\mathrm{lin}}$ on $r_{0}$ in Equation 28.

Figure 4 displays the dependence of ${\bar{C}}_{\mathrm{diff}}\left(s\right)$ for the two parameters $r_{0}/l$ and ω (given z is fixed). Yet, if we identify the truncation radius $r_{0}∼b$ with the lattice spacing, there should be a relationship between $r_{0}/l$ and ω . Recall the definitions $\omega =l_{B}/b$ and $l^{2}=\nu /\left(4\pi l_{B}\right)$ . The lattice spacing $b\approx \nu^{1/3}$ represents the size of the ions in the ionic liquid, typically on the order of a nanometer. If we identify the truncation radius $r_{0}=b$ with the lattice spacing, we obtain $r_{0}/l\approx \sqrt{4\pi }\times \sqrt{\omega }$ . Hence, the choice ω=1 (implying $l_{B}=b$ ) corresponds to $r_{0}/l\approx 3$ , which is displayed by the black curve in the right column of Figure 4. Further increasing the Bjerrum length (implying ω>1 ) yields larger values for $r_{0}/l$ . These cases are not shown in Figure 4 but are easily accessible through our analytic expression for ${\bar{C}}_{\mathrm{diff}}\left(s\right)={\bar{C}}_{\mathrm{diff}}^{\mathrm{lin}}+s^{2}\Omega /2$ .

**FIGURE 5:**
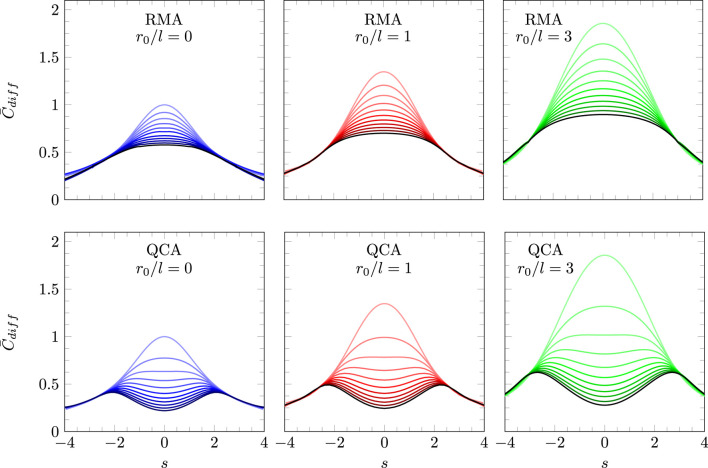
Scaled differential capacitance ${\bar{C}}_{\mathrm{diff}}$ as a function of the scaled surface charge density s based on RMA (upper three diagrams) and QCA (lower three diagrams). The lower three diagrams of Figures 4, 5 are identical. Each diagram shows 11 curves for ω=0 (uppermost curve) to ω=1 (black curve on the bottom), with ω changing in steps of 0.1. Diagrams refer to $r_{0}=0$ (left column, colored purple), $r_{0}=l$ (middle column, colored red), and $r_{0}=3l$ (right column, colored green). All curves are calculated for z=6 . All models are based on solving Equations 16, 17, yet with the relationship η(Ψ) emerging from Equation 38 for the upper three diagrams and from Equation 13 for the lower three diagrams.

Figure 5 shows a comparison of our model with and without accounting for electrostatic correlations between ion pairs. The lower three diagrams in Figure 5 reproduce the lower three diagrams of Figure 4, calculated for z=6 and using QCA where ion pair correlations are accounted for. The upper three diagrams show analogous results, yet in the absence of ion pair correlations, employing RMA, as introduced in Section 3.3.4. We have verified the agreement of the numerical results for RMA (upper three diagrams) in the region of small |s| with the analytic expression ${\bar{C}}_{\mathrm{diff}}\left(s\right)={\bar{C}}_{\mathrm{diff}}^{\mathrm{lin}}+s^{2}\Omega /2$ specified in Equation 39. As already pointed out, RMA does not predict a transition from a bell-shaped to a camel-shaped profile of the differential capacitance as the strength of the electrostatic interaction $\omega =l_{B}/b$ is increased. Hence, we conclude that electrostatic correlations rather than charge discreteness or excluded volume interactions cause camel-shape profiles of ${\bar{C}}_{\mathrm{diff}}\left(s\right)$ . Starting from the classical mean-field approach, ${\bar{C}}_{\mathrm{diff}}=\sqrt{1-e^{-s^{2}}}/|s|$ , displayed by the uppermost purple line on the left two diagrams in Figure 5, and introducing charge discreteness without accounting for electrostatic correlations corresponds to transitioning to the black curve on the right top diagram of Figure 5: the curve widens but without qualitatively changing its shape.

Because the presence of the bell-to-camel shape transition is independent of $r_{0}$ , the reason of its existence for a compact, solvent-free ionic liquid reflects the role of electrostatic ion–ion correlations. Correlated clusters of ions increase the decay length of the potential $\Psi_{\mathrm{lin}}\left(x\right)$ , as shown in Figure 3. Hence, if the system of electrode and diffuse layer of counterions is represented by an effective parallel-plate capacitor, the distance between the capacitor plates is larger in the presence of electrostatic ion–ion correlations. The correspondingly smaller value of ${\bar{C}}_{\mathrm{diff}}^{\mathrm{lin}}$ is consistent with the existence of a transition from the bell-shaped to a camel-shaped profile of ${\bar{C}}_{\mathrm{diff}}$ when electrostatic ion–ion correlations are accounted for.

## 4 Conclusion

The classical lattice-based mean-field theory for a densely packed, solvent-free ionic liquid employs continuum electrostatics and ignores ion–ion correlations, leading to a bell-shaped profile according to ${\bar{C}}_{\mathrm{diff}}=\sqrt{1-e^{-s^{2}}}/|s|$ for the scaled differential capacitance as a function of the scaled charge density s . The present work is an attempt to analyze how ${\bar{C}}_{\mathrm{diff}}$ changes if charge discreteness and electrostatic correlations between ion pairs are accounted for. To account for charge discreteness, we employ a truncated Coulomb potential (shown in Figure 1) to model the long-range part of the electrostatic interactions but exclude those of a given ion with its nearest neighbors. These short-range interactions are modeled explicitly as interactions between individual point charges. Electrostatic correlations between pairs of neighboring ions are accounted for using the quasi-chemical approximation (QCA) approach. Downing et al. (2018b) included QCA similarly to the present approach but without truncating the Coulomb potential, which led to a double counting of electrostatic interactions between nearest neighbor ions. The present work is no longer subject to this inconsistency. We emphasize that our approach is still subject to approximations: the truncation of the Coulomb potential is modeled only on the level of fourth-order electrostatics ( $A_{1}$ in Equation 7), and QCA ignores correlations of higher order and between pairs of ions separated by distances larger than one lattice spacing. Yet, these approximations can, in principle, be overcome by considering sixth- or higher-order electrostatics ( $A_{2}$ or higher in Equation 7) and by incorporating larger clusters into QCA (Bossa et al., 2015).

Our approximation of the full self-consistency relationship, AΨ=−η(Ψ)/ν , by the fourth-order differential equation, $A_{1}\Psi =-\eta \left(\Psi \right)/\nu$ , renders our model structurally equivalent to the BSK model (Bazant et al., 2011). An important aspect of our study includes the boundary conditions associated with that equation (Equation 17), which emerge as part of the functional minimization of the free energy (Equations 12, 19) and differ from previously proposed sets of boundary conditions (Bazant et al., 2011; Gupta et al., 2020b; de Souza and Bazant, 2020; Bossa and May, 2020).

A major result of our study is analytic expressions for the coefficients ${\bar{C}}_{\mathrm{diff}}^{\mathrm{lin}}$ and Ω in an expansion of ${\bar{C}}_{\mathrm{diff}}\left(s\right)={\bar{C}}_{\mathrm{diff}}^{\mathrm{lin}}+s^{2}\Omega /2$ up to quadratic order in s . We have obtained ${\bar{C}}_{\mathrm{diff}}^{\mathrm{lin}}$ from solving the linearized model and Ω from performing a perturbation approach into the non-linear regime. The quadratic expression of ${\bar{C}}_{\mathrm{diff}}\left(s\right)$ reveals the existence of a transition from a bell-shaped to a camel-shaped profile of the differential capacitance as a function of the electrostatic interaction strength ω between neighboring ions. However, such a transition exists only in the presence of electrostatic correlations, not in their absence. Hence, we conclude that electrostatic correlations between ion pairs are able to turn the bell-shaped profile predicted by mean-field theory, ${\bar{C}}_{\mathrm{diff}}=\sqrt{1-e^{-s^{2}}}/|s|$ , into a camel-shaped profile. Ion discreteness, on the other hand, enhances the magnitude but does not qualitatively alter the profile of ${\bar{C}}_{\mathrm{diff}}\left(s\right)$ . Our analytic predictions agree with numerical calculations of the full profiles for ${\bar{C}}_{\mathrm{diff}}\left(s\right)$ , i.e., beyond the quadratic regime, that we have presented in Figures 4, 5.

## Data Availability

The original contributions presented in the study are included in the article/supplementary material; further inquiries can be directed to the corresponding author.
